# What do patients and clinicians think about continuity in general practice in England? a qualitative study

**DOI:** 10.3399/BJGP.2025.0323

**Published:** 2025-12-16

**Authors:** Patrick Burch, Stephen Iles, Aaron Poppleton, Kath Checkland, Sarah Skyrme

**Affiliations:** 1 Centre for Primary Care and Health Services Research, University of Manchester, Manchester, UK; 2 Cornishway Group Practice, Manchester, UK; 3 School of Medicine, University of Keele, Keele, UK

**Keywords:** access to health care, continuity, general practice, patients

## Abstract

**Background:**

Relational continuity — an ongoing therapeutic relationship between a patient and a clinician — has long been a hallmark of general practice. However, its prevalence in England has declined over the past decade amid increasing demand, workforce shortages, and structural changes in primary care delivery.

**Aim:**

To explore patient and clinician views on continuity in general practice, and understand the factors influencing these perspectives.

**Design and setting:**

A qualitative study using interviews and focus groups conducted in England between July 2024 and February 2025.

**Method:**

Semi-structured interviews were conducted with 17 primary care staff, and six focus groups were held with 40 patients. Staff were recruited through team contacts and snowballing. Patients were recruited through adverts in general practices. Inductive thematic analysis was informed by theoretical models of continuity, Reeve and Byng’s United Model of Generalism, and the biopsychosocial model of care. Data were coded using NVivo software.

**Results:**

Four themes were identified: many patients lack experience of continuity and struggle to understand its value; patients and clinicians often prioritise other elements of care, such as quick access, over continuity; views on the value of continuity are shaped by beliefs about the role of general practice; and there is scepticism about the feasibility of restoring continuity within current system constraints.

**Conclusion:**

We may be reaching a tipping point, whereby a critical mass of patients views general practice solely as a method of accessing biomedical services from whichever staff member is available. If we want to improve continuity, we need to act before changes in attitudes and care delivery make change an impossibility.

## How this fits in

Continuity of care has long been considered central to general practice but, in a post-COVID-19 context, its decline in England has gone largely unexamined from both patient and clinician perspectives. Previous research highlighted the benefits of continuity, but often assumed that patients and clinicians valued it uniformly. This study shows that many patients now lack experience of continuity and, therefore, struggle to appreciate its benefits, whereas clinicians acknowledge its value, but doubt its feasibility. These insights are crucial for clinicians and policymakers who are seeking to promote continuity in a system in which structural and cultural shifts may already be undermining its relevance.

## Introduction

### Continuity in theory

Continuity of care has long been considered a key component of general practice, with the concept of the ‘family doctor’ being a culturally understood notion that people recognise.^1^ General practice has traditionally been seen as embodying the concept of a holistic biopsychosocial approach to medicine. In contrast with the biomedical model, it involves understanding the patient and their experience, as well as any underlying disease processes.^2^ Continuity between a patient and doctor can facilitate this approach, bring the goals of the patient and doctor into closer alignment, and reduce the unnecessary medicalisation of symptoms;^3,4^ this may partly explain why patient–doctor continuity is associated with multiple positive outcomes.^5–7^

Continuity of care can be categorised into three types:

- the ongoing relationship between a clinician and patient, which is *relational*;
- patient data that are recorded and stored, which provide *informational continuity*; and
- *management continuity*, which is coherence and seamlessness in the delivery of care between different healthcare providers.^8^

Although we acknowledge the importance of other forms of continuity in modern general practice, relational continuity between a clinician and patient has traditionally been seen as fundamental to the specialty and is more robustly linked to positive outcomes for the patient, clinician, and healthcare system in ways that other forms are not.^7^ In this article, we use the term ‘continuity’ to mean relational continuity — namely, an ongoing relationship between a patient and a doctor.

### Continuity in practice

Levels of primary care continuity in England and other high-income countries have been falling for more than a decade,^9,10^ and models of primary care have drifted towards a biomedical model focused on providing episodic care for acute problems.^11^ A lack of primary care continuity is also a problem in many lower- and middle-income countries.^12^ In England, increased demand for appointments^13^ and a reduced supply of GPs^14^ are likely to have contributed to this decline, as well as contributing to increased waiting times for appointments and reduced patient satisfaction.^9^ The policy and contractual response to this has further exacerbated the problem; the focus has been on speed of access over other elements of care,^15^ alongside a contract (the Quality and Outcomes Framework [QOF]) that is centred around the delivery of biomedical care.^2^

Policies to improve access, such as extended-access initiatives, have often had the unintended consequence of reducing continuity;^11,16^ likewise, introducing a broader range of staff into general practice has increased appointment capacity, but can also reduce continuity with a GP.^16,17^ The drift away from person-centred longitudinal care with a GP was exacerbated by a new GP contract in 2019, which prioritised access to *any* professional over GP-focused continuity, providing funding that was ringfenced to employ a range of non-GP clinicians and further deviating from the traditional GP model.^18^ Policy is continuing to push in this direction, with a recent report^19^ — the recommendations of which are now being implemented in several areas — suggesting offering separate ‘urgent’ and ‘routine’ services to patients accessing general practice. At the same time, GP workload has increased, GPs are working fewer days in clinical provision within routine general practice, and they have been leaving the profession in increasing numbers.^20^ It seems possible that creating a system based on a task-based biomedical model to solve the problems of access and a lack of GPs, rather than focusing on longitudinal person-centred care, has generated a cycle of negative reinforcement and is contributing to GPs leaving the profession.^21^

Changing behaviours in wider society, along with patient values, are also likely to have played a role in the reduction of continuity. GP practices are becoming larger, and larger practices tend to have lower levels of continuity.^22^ If interactions in retail, banking, and dating have changed to become increasingly non-relationship based, transactional, and focused around a few large-scale providers, it is perhaps unsurprising that some general practices have followed suit.^16^ Many of these issues are also present in other countries, with struggles to meet patient demand, expanding provider sizes, and increased use of alternative primary care providers having been noted where continuity of care has been seen to decline.^10,23^

### Improving continuity

Despite the challenges outlined above, in England and internationally there is currently a policy focus on improving relational continuity of care.^12,24,25^ However, there is a lack of evidence on *how* to improve it, with a diverse range of schemes in different settings having been attempted.^26,27^ Several policy and practical conundrums that need to be considered when trying to improve continuity exist, including:

- Should we be trying to provide continuity for everybody, or is there a subset of patients likely to benefit more?
- What is the role of patient choice?
- What influences people’s opinions about continuity of care?
- What is the role of continuity in GP job satisfaction and retention?

There is an evidence gap around these issues. Continuity has been shown to be particularly valued by certain patient groups, including older individuals and those with chronic illnesses.^28–30^ However, patient preferences for relational continuity are not uniform: patients may seek relational continuity for some issues but not others, and these preferences may evolve over time.^31^ Discrete-choice experiments — although context dependent — generally suggest that patients prioritise speed and efficiency for urgent health issues, with continuity of care being highly valued in other circumstances.^32,33^

Patient perceptions about primary care are impacted by complex shifts in policies, changing work patterns,^34^ and incremental modifications to appointment systems that confuse patients.^16,35^ Existing research suggests that GPs are highly supportive of continuity,^36^ but most of the research into patient and GP perceptions of continuity was carried out more than a decade ago. It is not known what patients and clinicians currently think about continuity and we do not know what factors influence their feelings. This article represents a first stage in trying to unpick some of these policy conundrums. The aims of the study were to understand GP and patient experiences and opinions, and to try to examine the current situation from both sides to better understand what might need to change. In the study, we examined three research questions:

- What value do GPs and patients place on continuity of care?
- What factors seem to be driving these opinions?
- What might need to change to realise the benefits of continuity of care?

## Method

This was a qualitative study using focus groups and one-to-one interviews, which took place between July 2024 and February 2025 in England.

### Sampling and recruitment

Primary care staff were recruited through purposeful sampling, based on known contacts from the lead author’s role as a GP and academic researcher, and through snowballing. As we sampled purposefully for maximum variation of experience and views, we did not interview all staff who agreed to take part. In total, 17 HCPs were recruited, characteristics for whom are summarised in Table 1. Adult patients (whose characteristics are outlined in Table 2) were recruited through advertisements placed in GP surgeries in Greater Manchester, the North East of England, and North Cumbria; contact details for the study researchers were provided and patients made first contact if they were interested in taking part.

**Table 1.** Characteristics of GP and healthcare staff participants

Role	Sex	Ethnic background	Age, years	Any training outside of UK?	Years of experience as a GP	Deprivation quintile of practice^a^	Practice size, patients	Area in England	Self-described availability of patient appointments
Salaried GP	Female	White British	36–45	No	11–15	1	20 000	Tyne and Wear	Urgent on-the-day or a few weeks’ wait
Salaried GP	Female	White British	36–45	No	6–10	1	10 000–12 000	Tyne and Wear	Urgent on-the-day or a 2-week wait
Salaried GP	Female	White British	36–45	No	6–10	3	8000–10 000	North Derbyshire	Majority of appointments are book-on-the-day
Salaried GP	Female	White American	36–45	Yes	6–10	1	8 000–10 000	Manchester	All requests triaged on the day with adequate access to same-day as well as delayed appointments
GP partner	Male	White British	36–45	No	11–15	3	20 000	Cumbria	Urgent on-the-day or a 4-week wait
Salaried GP	Female	White British	46–59	No	>20	3	10 000–12 000	North Derbyshire	1–2 weeks
Salaried GP	Female	White British	26–35	No	2–5	3	10 000–12 000	Tyne and Wear	Huge on-the-day capacity and reasonable next-week capacity
GP partner/primary care network/ clinical director/	Male	White British	36–45	No	11–15	3	6000–8000	Derby	Do very best to have good access but imagine 3 or 4 weeks in advance
GP partner	Male	British Asian	36–45	No	11–15	3	12 000–15 000	Tyne and Wear	2–3-week wait, large number of on-the-day appointments
Salaried GP	Male	British Asian	60–70	No	>20	1	10 000–12 000	Tyne and Wear	Not stated
GP partner	Female	White British	36–45	No	6–10	4	6000–8000	Cumbria	Try to see everybody on the day
GP partner	Female	White British	60–70	No	>20	3	12 000–15 000	Tyne and Wear	A lot of on-the-day with a roughly 3-week wait for routine
GP registrar	Male	African	46–59	Yes	<2	1	12 000–15 000	Lancashire	Not stated
Advanced nurse practitioner	Female	White British	60–70	No	n/a	n/a	Worked for area home visiting team	North Derbyshire	n/a
Advanced nurse practitioner	Female	White British	46–59	No	n/a	n/a	Worked across >1 practice	Manchester	n/a
Practice manager	Female	White British	36–45	No	n/a	3	10 000–12 000	North Derbyshire	Roughly 3-week wait for routine appointment with a large on-the-day capacity
Assistant director, integrated care board	Female	White British	36–45	No	n/a	n/a	n/a	Midlands	n/a

^a^Using the Index of Multiple Deprivation.

**Table 2.** Characteristics of patient participants

Category	Focus group participants, *n*
**Total**	40
**Gender**	
Female	33
Male	7
**Age, years**	
18–25	3
26–35	7
36–45	6
46–59	7
60–69	8
70–79	3
80	6
**Health conditions,** ***n***	
0	11
1–3	17
≥4	12
**Ethnicity**	
White British	28
Not White British	12
**Employment status**	
Full-time employment	9
Part-time employment	5
Full-time student	4
Student/part-time employment	4
Not in paid employment	7
Retired	10
Did not state	1
**Deprivation quintile by postcode^a^**	
1 (most deprivation)	12
2	3
3	7
4	5
5 (least deprivation)	3
Did not disclose postcode	10

^a^Using the Index of Multiple Deprivation.

### Data collection and data analysis

Semi-structured interviews were held online using video-conferencing software, face-to-face, or via telephone. Six focus groups were held with patients: two were held in person at community centres and four were conducted via video-conferencing software. Interviews lasted 23–53 minutes, and the focus groups lasted ~1.5 hours. The interview/focus group topic discussion guide was developed through consultation with the study’s patient and public involvement and engagement (PPIE) group (Supplementary Information S1), which also provided ongoing feedback and critique on the resultant findings. All interviews and focus groups were audio-recorded and professionally transcribed; audio-recorded consent was obtained from all participants prior to data collection.

Most of the GP and health staff interviews were conducted by the first author, an academic GP, but four were conducted by the last author, who is an experienced qualitative researcher. Three focus groups were led by the second author, and three were co-led by the first and last authors. Transcripts were anonymised then uploaded to NVivo 12 Plus software; systematic review and checking against audio recordings was carried out to ensure clarity of meaning.

We analysed the data from the first two focus groups and first three interviews using inductive thematic analysis. This showed that clinicians and patients held a diverse range of views about continuity. Rather than just describe these views, we discussed in the research team and with our PPIE group why participants may hold these views. This led to detailed engagement with theories about the nature of general practice, the biopsychosocial model, and Reeve and Byng’s integrated and interpretive model of primary care.^2,8,37^ Following this, we developed an initial coding framework. Initial coding topics included:

- limited experience of continuity;
- biopsychosocial — biomedical; and
- front desk — barrier/facilitator to continuity.

Although being open to new data and inductive analysis, our analytical strategy then became more abductive as we used our initial findings and readings of relevant theory to test our emerging findings with participants. A one-page sheet of discussion points was devised and sent to participants prior to attending the final three focus groups to direct discussions in the available time. We purposefully sought out views and data that would challenge our emerging analysis. Emergent themes were discussed and refined with the PPIE group, colleagues who have expertise in primary care delivery and research, and the research team, as part of an iterative, collaborative process of analysis. Reflexivity was integral to the analytical process. As reflexive researchers, we actively sought out robust challenges to our analysis of the nature of general practice and doctor–patient relationships. This came from our PPIE contributors as well as the wider research team.

## Results

Our results are presented in relation to the four principle themes that were constructed during the analysis. The participants are identified by the letters ‘PL’ (clinician or NHS administration/managerial staff) and PFG (patient focus group). All responses have been anonymised.

### Theme 1: how do you know what continuity is if you have never had it?

Among the patient participants, there was a split between those who had experienced continuity and those who had not. For those who had experienced it, or who recognised that they needed it, there was generally a preference for continuity; among those with limited or no experience, however, few expressed a preference. Nearly all patients indicated the importance of informational continuity, but felt that relational continuity was not necessary to achieve this:

*‘So I’ve experienced both, in childhood having the same doctor … and in adulthood not seeing the same doctor, and for me that doesn’t bother me, but what does bother me is the consistency in communication within GP services. So I don’t mind seeing a different person as long as those people are all consistent in the way that they read up on the notes …’* (PFG5)

Many patients, including this patient in their early 60s, had no expectation of receiving continuity:

*‘I have never been under the impression that I had my own GP. I thought I was just within a medical practice, so I just took whatever appointment. I wouldn’t have said I want to see such-and-such a GP.’* (PFG2)

Patients were almost universal in identifying issues with uncoordinated and fragmented care in the NHS:

*‘It’s just like you’ve got to see one person and they can say, “Go see this person”, and that person passes you to somebody else. It’s like they’re just passing the buck onto somebody else.’* (PFG1)

However, they did not necessarily see relational continuity with a GP as a potential antidote to this.

Discussing the pros and cons of continuity was a theoretical exercise for many of the patients to whom we spoke, who had no experience of continuity with primary care clinicians. However, some patients with little of no GP continuity did report continuity with other primary care professionals.

*‘I'm thirty, you know, I've never seen the same GP all the time. I have moved around a lot, but even when I stayed in the same place for a number of years, I've always seen different people. Apart from nurses, I've been on, kind of, long term contraception of various forms for a long time. And so, at the moment, I go in every twelve weeks, and I usually see the same nurse. And that, kind of, puts me at ease …’* (PFG4)

An illustrative example of relational continuity and trust between a GP and a patient with complex needs was shared with one of the focus groups. A participant, who had not experienced continuity, observed how this example brought new insight to the discussion, helping them imagine what continuity might mean:

*‘I think it’s interesting to think about it from what the GP had said about that patient who listened to him more, which is nothing that I’d considered … having that relationship with a GP your whole life, trusting them, knowing that they know.’* (PFG6)

In contrast with those who felt that relational continuity was largely irrelevant to them were patients who always regarded continuity as relevant for all patients. This included an individual who had experienced serious illness; they commented that:

*‘We need continuity for everybody. I need to know, if I go with a cough, and that’s not cleared with antibiotics in two weeks’ time, I can go to the same GP. I don't think there’s anything that is minor, because we don't know where complex is going to come in. I wasn’t expecting, at forty-two, to be diagnosed with breast cancer.’* (PFG3)

Several patients who were not born in England were able to compare their recent healthcare experiences against those in their country of origin, and often lamented the lack of continuity in England:

*‘I'm* [identifier of nationality] *… I used to see, always, the same GP. I really don't know the ins and outs of why that is difficult here … I was completely baffled when I realised that that wasn’t the case here.’* (PFG3)

GPs who had been practising for 10 years or more recognised the decline in continuity and generally saw it as a bad thing:

*‘The climate has changed significantly to what it used to be thirty years ago, where someone saw the same doctor and that doctor just worked out what was going on with them, and they could just keep them on the straight and narrow if you like.’* (PL6, GP)

Less experienced GPs understood that continuity was considered a core part of general practice and recognised its potential benefits, but several had limited experience of delivering it. Their experience of continuity was often sporadic, and delivering care to patients they knew and with whom they were familiar was the exception rather than the rule. They acknowledged that this could result in fragmented care, difficulties in decision making, and problems with training:

*‘That’s one of the reasons why GP training’s very, very stressful now — because the easy bits that can easily take off the stress from you have been taken away already, either by the nurse … our colleagues who are nurse practitioners or the PAs* [physicians assistants]*, so in a way we’re already seeing less and less of such patients … it can remove the variety from the GP and make the work much more daunting.’* (PL14, GP registrar)

### Theme 2: continuity versus other priorities

This theme explores the importance and prioritisation of continuity for patients and clinicians. Many, but not all, patients and clinicians wanted relational continuity but struggled to get it; other patients battled to get any form of access and, consequently, prioritised this over continuity:

*‘I'm forty-seven, and I’d say I’ve easily not had any GP continuity in probably about thirty years. And to be honest, the issue is just trying to get an appointment, which is generally, absolutely impossible. You have to phone at eight o’clock, trying to get an on-the-day appointment, pre-bookables are gone, there’s never anything online. It’s an absolute nightmare, and I’d say it’s been that way for at least the last eight plus years. It’s quite shocking.’* (PFG4)

Some patients did not regard themselves as needing continuity, but preferred rapid access to appointments when necessary:

*‘I think I would say, actually, I don’t need that* [continuity]*; other people will take priority, that’s fine, I’m happy to see someone quicker.’* (PFG5)

However, there were patients who wanted continuity and were often frustrated when they were unable to get it:

*‘I do have a couple of long-term health conditions and, to be quite honest, I would rather see a named doctor that specifically knows what’s wrong with me. And with me having a learning disability, I find it very frustrating that I can’t see that certain doctor or get an appointment to see the same doctor.’* (PFG5)

Several patients objected to the idea of being ‘assigned’ to a particular doctor and seeing a different GP was recognised as beneficial in some cases, supplanting continuity as a mechanism for good care:

*‘One doctor could have missed something, and another doctor might actually spot something or do a different test. So, you could be actually improving your healthcare by seeing different doctors.’* (PFG2)

Although GPs mostly thought improving continuity was important, several were concerned that there were more pressing priorities. Access to appointments and issues with estates were more important for one GP, who stated:

*‘Access for me would trump continuity, I think. But they’d be pretty close actually. I don’t know actually. ’Cause I always think the issue is access. You know, our big issue is this four-week wait for a routine appointment and not enough capacity. That’s probably our bigger issue. Then people get on the urgent care on the day because they say, “wWell I can’t wait four weeks.” Then it’s, “oh, well, I’ll put you on the urgent care”, and it ends up with a doctor who’s not their named doctor or in their team. Then that doctor normally says, “book a routine appointment” cause whatever it is, it can wait that long. So it kind of comes together, doesn’t it? I don’t know how you sort one without the other … If I was in charge of all the money, I wouldn’t just focus on continuity. There’s lots, you know, estates [buildings, physical infrastructure], there’s massive things that it wouldn’t just be continuity. Would sorting out all the continuity make my life easier as a GP? It probably would, but I don’t know if it would vastly make it better, is my honest answer.’* (PL5, GP)

### Theme 3: what’s the point of continuity? Retention of information versus relationship building and holistic care

All the clinicians interviewed, as well as the majority of patients, recognised that there were particular patients for whom continuity would likely be beneficial. There were, however, very different ideas about which patients should receive continuity; this could partially be explained by the participants’ attitudes regarding the purpose of general practice. With both patients and clinicians, there appeared to be a spectrum of views. At one end were those who thought of general practice as a series of discrete biomedical transactional encounters for which, if a clinician was competent, only continuity of information and management were important. This view appeared to be held by several patients, and was not necessarily related to their age or health condition, as illustrated by this quotation from a patient aged in their 70s with chronic health problems:

*‘It doesn’t matter to me who I see, but it’s having them know what you were there for before and why you're there now.’* (PFG5)

At the other end of the spectrum were those (mostly more experienced GPs) who felt that most consultations involved more than biological facts, and that a longitudinal relationship-based biopsychosocial approach was almost always relevant:

Interviewer: *‘*[What] *about the concept of certain groups of patients that arguably need continuity less?* [They] *get seen by anybody, maybe outside* [of] *the practice, and the ones that need it get seen by the GPs?’*Participant: *‘Yes, first of all, I’ll say I’m aware of that concept and, secondly, I think it’s expressed by people who don’t really understand what goes on in the consultation, because they imagine the consultation as a technical issue or diagnosis or prescription, and you know and I know it’s a lot more complex than that …’* (PL10, GP)

Whether or not patients wanted continuity, they all wanted doctors who had ‘softer’ skills, such as listening, showing compassion, and viewing them holistically:

*‘… sometimes you go see one GP and they're quite dismissive and then you don’t feel as confident because you think, “OK, well, maybe what I had to say wasn’t important.” But then you leave that appointment feeling, “Oh, who else do I turn to if I can't speak to my GP?”’* (PFG5)

*‘I do also feel that they shouldn’t be so brutal with you, or* [that they should] *have that what they used to call the bedside manner where they were nice to you, they were kind to you, because you can go in there scared to discuss a problem, but then you can be made to feel ten times worse by their attitude towards you.’* (PFG 1)

All GPs acknowledged the benefits of seeing the same patients and building a relationship and knowledge about them:

*‘I have … a lot of background information, which I think is useful. It’s useful in terms of making decisions and it’s useful in terms of building trust, so it’s a very real use, it’s not just a nebulous theoretical use. It actually comes into play almost every day of my working life, and I do struggle to understand how that’s going to be possible in the future without the degree of continuity.’* (PL10, GP)

Several GPs described the hard-to-measure benefits of continuity, which develop from knowing their patients, from engendering trust and mutual understanding. This was described as bringing a sense of satisfaction to GPs’ working lives and developing a sense of ownership of their patients’ care that was built through repeat consultations with the same patients:

*‘… a bit of human contact I think goes a long way for our own morale. The patients really do appreciate not starting over, I find almost, sometimes,* [that] *continuity is all I can offer someone who’s got a complex history, abuse, chronic pain.’* (PL16, GP)

This attitude was shared by patients who had also experienced the less tangible benefits of continuity:

*‘But I think, seeing the same person was really pivotal, because he knew from the second appointment, that we’ve already tried all this other stuff that didn’t work … So, let’s just immediately proceed to the next phase … So, whether it’s episodic or long term, I think it is important to see the same person. Because it does build trust, it builds confidence that this person that you’ve been seeing knows what you’re talking about, they’ve listened to you.’* (PFG3)

### Theme 4: resigned pragmatism — acceptance that the era of the family doctor is over?

Although the potential benefits of continuity were generally acknowledged by many patients and clinicians, there were few participants who thought that efforts to improve continuity would be successful or desirable without other changes. Findings related to Theme 2 demonstrated how issues of capacity and access were often more important to patients and GPs than continuity. Of the staff with whom we spoke, even the most ardent supporters of continuity accepted that it could be difficult to deliver with an increasingly multidisciplinary workforce and when more than half of all general practice appointments are delivered by non-GP staff.^38^ Staff felt that the move towards larger-scale multiprovider, multidisciplinary general practice was not something that was likely to change. Some clinicians lamented the end of continuity, and some saw it as part of the general decline — and potential end — of general practice:

Participant: *‘So, I think for me, as a person, the greatest sort of satisfaction of general practice, is actually the continuity of care.’*Interviewer: *‘If a lot of that was taken away from you, how would you see your job?’*Participant: *‘Well, I think we already think that general practice … as it’s known, is already dead … if they want to formalise that and just, like, writing a death certificate … killing such an approach to care that has been proven to work over time.’* (PL14, GP registrar)

However, some GPs reported that they were able to provide continuity to many of their patients. This was facilitated through having continuity as an organisational priority, with systems in place that facilitated continuity (for example, having named doctors or having flagged ‘continuity’ patients):

*‘So we’ve got follow-up slots on every single clinic that we ever do, and they’re not for patients to come in … So we encourage personal follow-up, we’re encouraged to put work in a place that is visible and you’re reminded to review people.’* (PL7, GP)

An NHS manager mentioned a focus on delivering continuity for particular patients:

*‘So, I think it is important, understanding which of the population needs that continuity, why it’s important, not just because they like it* [but] *because, actually, it’s needed to manage their condition, and then you can just use an alternative professional to get what they need on the day or, you know what I mean, low-complexity patients.’* (PL4, NHS manager)

This was also echoed by some GPs. However, there were several GPs who expressed concern that, if their role was changed and they were ‘forced’ to deliver continuity only for those patients deemed to require it because of having complex needs, this could lead to their job being more difficult:

*‘There’s a lot of miserable doctors out there at the moment, and GPs, for obvious reasons. I think if you leave the GPs with all complex, frail patients every day or whatever it might be that* [that] *also is not healthy. It’s nice to have a bit of a break in your list to help, a simple UTI* [urinary tract infection] *or a simple cough, a simple cold as well.’* (PL6, GP)

## Discussion

### Summary

The value placed on relational continuity of care appeared to be directly related to patients’ experience of it. Many patients had little or no experience of receiving relational continuity, and some GPs had limited experience of delivering it. Difficulties accessing any form of general practice had led many patients to prioritise simply getting an appointment over trying to get an appointment with a particular clinician. Most GPs, even those who had little experience of relational continuity, understood the less tangible benefits it can bring and most said they would like to provide more relational continuity to their patients. However, it was recognised that, although some clinicians might be able to provide continuity, and some practices had organisational cultures and processes to facilitate it, it can be difficult to deliver in contemporary general practice. Most GPs did not foresee a return to a ‘one-patient, one-doctor’ model of care.

The rush to secure an appointment with *any* clinician has superseded many patients’ expectations of continuity, leading to patient behaviour that is influenced by how care is delivered.^39^ This, in turn, puts pressure on primary care because patients who might previously have been prepared to wait to see the same GP may no longer have this option available to them. Our findings suggest that patients appear to want the benefits of continuity, without wanting the potential drawbacks, such as difficulty with access, waiting, or relying on the expertise of just one clinician. Patients understand that a relationship of trust may develop with time, but there is probably a lack of patient understanding as to how clinicians use time and repeated consultations to form a picture of a patient that can better enable them to practise more efficient, holistic, patient-focused care.

### Strengths and limitations

This qualitative study used purposeful sampling to understand a wide range of clinician and patient views. It is the first study that we know of to explore this topic in depth with patients and clinicians in post-COVID-19 general practice in England. We sampled patients and clinicians for a wide range of characteristics that we thought might influence their attitudes towards continuity. We sampled for information power,^40^ rather than to represent characteristics of any underlying population, but it should be noted that our GP sample had a predominance of GPs from larger-than-average practices and our patient sample had a disproportionately high number of female participants. Although we did hear a diverse range of views, it is not possible to draw reliable conclusions about how prevalent different attitudes towards continuity are, based on this methodology. Attitudes towards continuity reflect the pressures and current realities of general practice in England and, although this allowed the generation of theory that may have a more universal application, the findings could well be different in other healthcare systems.

### Comparison with existing literature

Continuity of care is associated with improved outcomes for patients, doctors, and the healthcare system,^6,7^ and it is known that patient and clinician views on continuity are diverse and often nuanced.^30,31^ This study adds to this literature by examining attitudes in a system with low continuity levels, in which many patients struggle to access any form of appointment. ^39^ In common with other literature about patient experience,^30^ our results show that patients want informational continuity and a clinician they can trust; they also often spoke of a preference for a holistic approach to their care. Although these can be delivered through a one-off consultation and are espoused in the concept of relationship-based care,^41^ evidence suggests that relational continuity is a key mechanism through which these aspects of the consultation can be achieved.^4,42,43^ All our patients spoke of their desire for informational continuity; however, other qualitative work has suggested that GPs think patient notes are insufficient in conveying the subjective, broad, and detailed information arising from consultations.^44^ This may be further exacerbated in England by giving patients online access to their medical record, which may have decreased the amount of soft or impressionistic information that GPs record.^45^ An international survey of GPs carried out in 2003^46^ showed the high value that GPs placed on personal continuity and the belief that good informational and/or management continuity was not sufficient for ‘most patients’. Although some of the more experienced GPs in our sample agreed with this, many did not or reported that they would be unlikely to see a potential way of providing continuity to most of their patients within the current structures of general practice.

### Implications for research and practice

A lack of experienced continuity can make it difficult for patients to understand its more subtle benefits — aside from those of informational continuity — and, therefore, make them less likely to seek it out; potentially, then, they may be less likely to support changes to improve continuity at the expense of quicker access. The view that continuity was generally not needed for most patients was echoed by non-GP NHS staff and many patients. Although our research cannot judge the prevalence of such views in the wider population, the opinions of those people in our sample may reflect a gradual underlying shift in how GPs, NHS staff, and patients see the purpose and function of general practice in England. This, in turn, may reflect the shift away from a biopsychosocial model of general practice based on longitudinal relationships to a more guideline-driven multidisciplinary biomedical model. It is also possible that the acceptance of the loss of continuity we encountered in some of our sample is symptomatic of a wider societal shift, whereby multiple aspects of human interaction — such as accessibility, speed, choice, and use of IT — have increasingly replaced continuity with a known individual.

There have been several efforts to improve continuity and there is ongoing research examining the characteristics of practices with high levels of continuity and how they achieve it.^26,47^ We know there are factors associated with higher levels of continuity; some of these are easily amenable to interventions, while others are not. Smaller practices with higher proportions of GP-delivered appointments^38^ and a practice culture that values continuity^48^ are associated with better continuity, but are not easily modifiable factors. Ultimately, policymakers will need to decide whether they are prepared to make the significant structural, human resource, and financial changes to the delivery of general practice that are needed to successfully implement a model of general practice in which most patients have continuity with ‘their GP’. To do this, we will need to solve the problem of access.

Evidence suggests that better continuity of care is associated with reduced primary care usage,^49^ but patients are unable, or unprepared, to put up with long waiting times to get an appointment with the same GP. This negative cycle needs to be broken by investment in GPs and increasing the availability of GP appointments. We need to revitalise the idea of holistic, patient-focused care that is delivered by GPs with good communication skills. We also need to think about access in a more nuanced way, with practices prioritising individuals’ needs, rather than setting strict rules about access.^50^ We need GPs to be incentivised to provide continuity and consider which models of practice organisation can sustain a more person-centred approach.

The alternative to this is to try smaller-scale initiatives that fly in the face of prevailing policy shifts towards the large-scale delivery of multidisciplinary biomedical primary care. One option would be to focus on trying to provide continuity for those patients deemed to need it the most. However, this risks treating many patients with a standardised, more transactional biomedical approach to care that misses out on the known benefits of continuity. Many interactions in general practice are for single issues when a standardised approach can be taken;^37^ however, when a patient presents to general practice, it is often not easy to judge whether a standardised non-interpretive approach is required. If we focus continuity on selected patient groups, we need further research to be undertaken so we can understand which groups are most likely to benefit and how to select them. Whichever approach is adopted, the diverse general practice workforce involved in the delivery of primary care needs to be carefully considered. The focus of this article has been on GP–patient continuity, but patients reported benefits of continuity with other general practice HCPs, such as nurses and physiotherapists. More research needs to be done into the importance of continuity with non-GP clinicians, while also understanding how the skill mix in general practice can negatively affect GP continuity.

Attempts to improve continuity also need to incorporate patient choice. It is clear from our results that patients with positive experiences of continuity valued a trusting, patient-centred, therapeutic relationship. Continuity is only a proxy marker for this; it is the *nature* of the relationship between the doctor and the patient that is important, and it is possible that this, rather than a patient simply seeing the same person repeatedly, is the driver of many of the positive outcomes associated with continuity. Our patient participants and our PPIE group talked repeatedly about how they did not want to be ‘forced’ into continuity with a GP they may not want to see; this is important to remember if considering measures to improve continuity. At the same time, there is a risk that, by leaving the patient to choose whether they want continuity, patients who are more socioeconomically privileged will be the ones who will reap the benefits of continuity, further exacerbating health inequalities.^51^

There is ongoing research to try to understand how some practices achieve high levels of continuity^47^ and which mechanisms link continuity with better outcomes in which patient groups;^52^ it would be useful to understand, from a quantitative perspective, which groups of patients benefit most from continuity. Future research priorities in England will depend on whether the government decides to incorporate improving continuity into the GP contract or implement strategies for differential access to primary care based on demographic characteristics or perceived clinical need. Whether someone has (or provides) relational continuity is a subjective judgement. Regardless of the presence or absence of relational continuity, efforts should be made to maximise experienced continuity for all patients.^53^ The focus of this article has been around overarching patient and health professional views of continuity, rather than the means by which GPs can increase relational continuity in practice; this topic warrants further research.

Low patient satisfaction with access and patient reported unmet health needs, suggests that demand for general practice services exceeds supply in many parts of England.^9^ Under such pressure, and with an increasingly part-time, non-GP-based workforce, it is understandable that relational continuity with a GP is not seen as a priority — or even possible — for many patients and GPs. However, by accepting its decline, or focusing it on a narrow population only, we risk further cementing the shift towards a ‘one-size-fits-all’ biomedical approach to primary care. Although this may be effective for some groups of patients, much of the work that is done in general practice is biopsychosocial in nature and is most effectively carried out using an approach based on longitudinal relationships. Continuity between a doctor and patient makes this much easier.

This work highlights the benefits of continuity as experienced by GPs and patients, but warns that there are now many patients who have little, or no, experience of continuity; consequently, they often do not understand the potential benefits that it can bring and, therefore, have other priorities when accessing general practice. Given the current low rates of continuity experienced by patients, we may be reaching a tipping point whereby a critical mass of patients (and voters) view general practice solely as a method of accessing biomedical services from whichever staff member is available. If policymakers and professionals want to improve continuity, action must be taken before patients no longer care about it and before the structural and workforce changes in general practice are so far advanced as to make any meaningful change an impossibility.
